# Impairment of Methotrexate Transport Is Common in Osteosarcoma Tumor Samples

**DOI:** 10.1155/2011/834170

**Published:** 2010-12-22

**Authors:** Rebecca Sowers, Bethanne D. Wenzel, Condon Richardson, Paul A. Meyers, John H. Healey, Adam S. Levy, Richard Gorlick

**Affiliations:** ^1^Division of Hematology/Oncology, Department of Pediatrics, The Children's Hospital at Montefiore, The Albert Einstein College of Medicine of Yeshiva University, 3415 Bainbridge Avenue, Rosenthal Rm 300, Bronx, NY 10467, USA; ^2^Department of Pediatrics, Memorial Sloan-Kettering Cancer Center New York, NY 10065, USA; ^3^c/o Veterans Administration New York Harbor Healthcare System, Narrows Institute for Biomedical Research Brooklyn, NY 11209, USA; ^4^Charlotte Kimelman Cancer Institute, 9048 Sugar Estate, St. Thomas USVI 00802, British Virgin Islands; ^5^Division of Orthopaedic Surgery, Department of Surgery, Memorial Sloan-Kettering Cancer Center New York, NY 10065, USA

## Abstract

Osteosarcoma does not respond well to conventional dose methotrexate but does respond to high-dose methotrexate. Previous work has indicated that this resistance may be due to impaired transport of methotrexate across the cell membrane. In this study, the PT430 competitive displacement assay was adapted to evaluate methotrexate transport in 69 high-grade osteosarcoma tumor samples. All samples studied were shown to have relatively impaired methotrexate transport by PT430 assay. Ninety-nine percent of the samples had less than 20% PT430 displacement by methotrexate. Eighty-eight percent exhibited displacement by methotrexate at less than 50% of the displacement by trimetrexate. The high frequency of impaired transport suggests the presence of decreased functionality of the reduced folate carrier protein. The overwhelming presence of impaired transport may explain why methotrexate needs to be given in high doses to be effective in osteosarcoma therapy and suggests that reduced folate carrier-independent antifolates should be explored.

## 1. Introduction

Although osteosarcoma is uncommon among the general population, it is the most common primary malignant bone tumor in children and adolescents [1]. It is believed that osteosarcoma is not responsive to conventional dose methotrexate in contrast to high-dose methotrexate [2, 3]. Most current multi-agent treatment regimens include the administration of high-dose methotrexate with the combination regimens having a five-year disease-free survival rate of 60% or greater [1]. This requirement for methotrexate in high doses for effectiveness may be explained by an intrinsic resistance of osteosarcoma to transport the drug across the cell membrane. 

Methotrexate is a structural analog of folic acid and acts by binding and inhibiting dihydrofolate reductase (DHFR), a key enzyme required for intracellular folate metabolism [4]. Intracellular methotrexate undergoes polyglutamylation whereby the polyglutamylated methotrexate is preferentially retained in the cell and ultimately results in DHFR inhibition [5–7]. Resistance to methotrexate in model systems has been attributed to several causes including loss of or decreased reduced folate carrier (RFC) function [8], increased DHFR expression potentially as a result of gene amplification [9], and diminished intracellular retention of methotrexate secondary to decreased polyglutamylation [7]. Additionally, changes in downstream efflux pathways could affect the intracellular concentration of methotrexate [10].

Methotrexate can be transported by at least three routes: the folate receptors, the reduced folate carrier, and the proton coupled folate transporter [10]. The proton coupled folate transporter has optimal transport when in an acidic environment [10]. The folate receptors have a higher affinity for folic acid as compared with the reduced folates while the RFC has a higher affinity for reduced folates and methotrexate as compared with folic acid [3]. The RFC has an exponentially greater cycling rate than folate receptors. The role of folate receptors in antifolate transport may be relevant only when RFC function is quite low unless the antifolate in question has a particularly high affinity for the folate receptor or if the folate receptor is highly expressed [10]. 

Trimetrexate does not require the RFC for cell entry; however, limited clinical studies have been performed using trimetrexate for the treatment of pediatric solid tumors. Some studies have suggested that methotrexate transport defective cells are more sensitive to trimetrexate [11] and could potentially overcome methotrexate transport resistance [7].

Previous work has demonstrated that over 50% of osteosarcoma samples have at least one sequence alteration in the RFC [12]. Another study has shown decreased RFC mRNA expression occurs in 65% of osteosarcoma samples obtained at biopsy and in 50% of metastatic or recurrent samples [13]. The same study concluded that 10% of osteosarcoma samples have increased DHFR mRNA expression at time of biopsy and 62% of metastatic or recurrent samples have increased DHFR. PT430, a fluorescent lysine analog of methotrexate, competes with both methotrexate and trimetrexate for DHFR binding. Where PT430 is displaced by trimetrexate and not by methotrexate, the difference in displacement can be attributed to defective transport of methotrexate into the cell [14]. In this report the PT430 competitive displacement assay has been adapted to assess methotrexate transport in osteosarcoma.

## 2. Materials and Methods

### 2.1. Sample Collection

Osteosarcoma samples were collected at Memorial Sloan-Kettering Cancer Center between November 1997 and June 2001 after obtaining written informed consent in accordance with a biology study approved by the Memorial Hospital Institutional Review Board. Additional samples were collected as part of the Children's Oncology Group P9851 Osteosarcoma Biology Study also after obtaining written informed consent. All samples were confirmed to have a pathologic diagnosis of osteosarcoma.

### 2.2. Establishment of Short-Term Cell Cultures

Approximately 25 mg of fresh tumor were finely minced using a sterile scalpel. The minced tissue was incubated for at least two hours in 5 mLs of disaggregation media composed of MEM-alpha media, 20% FCS (HyClone, Logan, UT), 0.6% collagenase Type 2 (Worthington Biochemical, Lakewood, NJ), and 0.002% DNAseI (Promega, Madison, WI). After incubation, the slurry was passed through a 70 *μ*m cell strainer. The filtered solution was centrifuged at 200 ×g and the resulting cell pellet was resuspended in 20 mls of cell culture media (MEM-alpha media + 20% FCS + 1% pen-strep) and subsequently plated in a Corning T75 flask. Cells were passaged after reaching 80% confluence.

### 2.3. PT430 Competitive Displacement Assay

The PT430 competitive displacement assay was performed as previously described [8, 14] with the following modifications. The assay was performed with the osteosarcoma cells placed in suspension as described originally with the cells trypsinized to place them in suspension. The trypsinized cells were centrifuged at 200 xg and the resulting cell pellet was resuspended in 5 mLs of media. Cells were kept in suspension through the completion of the assay. Cells were incubated in 20 *μ*M PT430 for four hours at 37° instead of the referenced two hours. Methotrexate and trimetrexate were added to final concentrations of 1 *μ*M each. PT430 was synthesized and kindly provided by Dr. Joel Wright and Dr. Andre Rosowsky of the Dana-Farber Cancer Institute. Methotrexate and trimetrexate displacement were calculated relative to the peak uptake correcting all samples for autofluorescence as has been described previously.

### 2.4. GD2 Antibody Labeling

GD2 antibody labeling was performed to verify that the cell population consisted of osteosarcoma cells and not fibroblasts. 1 × 10^7^/ml cells were incubated with 100 *μ*g 3F8 mouse monoclonal antibody for 1 hour at 4 degrees C on a rotating platform. The 3F8 antibody is specific for the GD2 cell surface protein which is present on osteosarcoma cells but is absent on fibroblasts. Following incubation with the primary antibody, cells were washed twice with PBS and then incubated with 50 *μ*g of mouse IgG-FITC secondary antibody (Research Diagnostics Inc., Flanders, NJ). Secondary antibody incubation was for 15 minutes at 4 degrees C on a rotating platform. After incubation, cells were washed twice with PBS. The remaining pellet was resuspended in PBS and analyzed by flow cytometry. (The 3F8 antibody was kindly provided by Dr. N. K. Cheung, Memorial Sloan-Kettering Cancer Center).

## 3. Results

Tumor samples were obtained from 69 patients with confirmed diagnosis of osteosarcoma. Fifty of the 69 samples were obtained at time of biopsy, 11 samples were obtained at time of definitive surgery, and the remaining eight were obtained at the time of relapse or at the site of metastatic disease. GD2 antibody labeling of a subset of patient samples demonstrated that more than eighty-five percent of cells were osteosarcoma tumor cells (data not shown).

**Figure 1:**
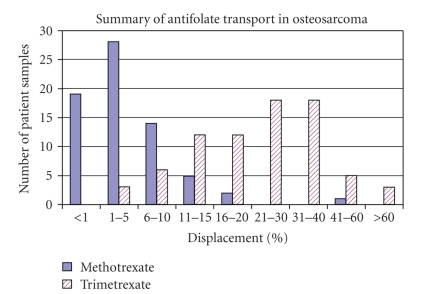
Summary of percentage PT430 displacement by methotrexate and trimetrexate in 69 osteosarcoma patient samples. Methotrexate displacement of PT430 was less than 20% in 99% of the samples.

PT430 displacement by methotrexate and trimetrexate in the 69 osteosarcoma patient samples is summarized in Table 1 and Figure 1. Sixty eight of 69 samples (99%) had less than 20% displacement by methotrexate with 17 samples evidencing a complete lack of PT430 displacement by methotrexate. In comparison, trimetrexate displacement of PT430 was measurable in all 69 patient samples. Displacement by methotrexate among the 69 samples ranged from 0% to 51% (mean = 4.9%); displacement by trimetrexate ranged from 3.3% to 86.7% (mean = 24.7%). 

In 60 of the 69 samples, displacement by methotrexate (MTX) was less than half the displacement by trimetrexate (TMTX) suggesting an RFC-mediated transport defect. This is represented by an MTX : TMTX differential displacement ratio where the percentage displacement by methotrexate is compared to the percentage displacement by trimetrexate (Table 1). In two of the 69 samples, PT430 displacement by methotrexate was greater than displacement by trimetrexate which may be related to P-glycoprotein expression. Nine samples had elevated peak PT430 accumulation signifying possible DHFR overexpression [8, 15].

All 50 biopsy samples exhibited less than 20% methotrexate displacement of PT430 (range = 0% to 19.3%; mean = 3.6%) with 16 samples displaying 0% displacement by methotrexate. Trimetrexate displacement of PT430 ranged from 3.3% to 59% (mean = 20.6%). Forty five of the 50 biopsy samples (90%) exhibited methotrexate displacement of PT430 at less than half of the trimetrexate displacement. The average MTX : TMTX differential displacement ratio of the biopsy samples was 0.24 indicating that, on average, methotrexate displacement of PT430 was only 24% of the PT430 amount that was displaced by trimetrexate. 

Of the 11 definitive surgery samples, 10 samples displayed less than 20% methotrexate displacement of PT430 (range = 0% to 51%; mean = 8.9%). One sample displayed 0% methotrexate displacement of PT430 and one sample displayed 51% methotrexate displacement. Trimetrexate displacement of PT430 ranged from 6.8% to 86.7% with an average of 32.8%. Nine of the 11 definitive surgery samples (82%) displayed methotrexate displacement of PT430 at less than half of the corresponding trimetrexate displacement with an average MTX : TMTX differential displacement ratio of 0.27.

All eight samples representative of metastatic disease or relapse had methotrexate displacement of less than 20% (range = 1.1% to 14.7%; mean = 7.4%). Six of the eight samples (75%) showed methotrexate displacement at less than half of trimetrexate displacement. Trimetrexate displacement for the eight samples ranged from 12.5% to 93.1%; mean = 38.7%. The average MTX:TMTX differential displacement ratio was 0.25.

## 4. Discussion

Methotrexate treatment is a valuable component of current treatment protocols for osteosarcoma [16–19]. Unfortunately, high-dose methotrexate therapy is associated with considerable costs and morbidity, although significant neurotoxicity and renal toxicity are rarely observed. The improved efficacy of high-dose methotrexate as compared to conventional dose methotrexate suggests that osteosarcoma may have intrinsic methotrexate resistance which can be overcome by achieving a high extracellular drug concentration [4, 10]. One possibility for intrinsic methotrexate resistance would be a transport defect. A clearer understanding of the relative methotrexate-resistance of osteosarcoma may direct better treatment strategies.

This study sought to determine whether the impaired cell entry of methotrexate is involved in the observed intrinsic resistance to methotrexate in osteosarcoma. When PT430 is displaced by trimetrexate and not by methotrexate, the difference in displacement can be attributed to defective transport of methotrexate into the cell [14]. Currently, there is no standard for what is considered functionally defective methotrexate transport in osteosarcoma. For the PT430 assays described in this study, methotrexate was added to a final concentration of 1 *μ*M. Of note, this is a clinically relevant methotrexate level, and typical plasma concentrations following high-dose methotrexate for osteosarcoma patients are <10 *μ*M at 24 hours and <1 *μ*M at 48 hours. In acute lymphocytic leukemia, defective transport has been defined as less than 40% displacement of PT430 by methotrexate [20]. A recent study investigating impairments in antifolate transport in retinoblastoma tumors suggested a reduced folate carrier protein defect was indicated in those samples whose PT430 displacement by methotrexate was less than half the displacement by trimetrexate [12].

In this study, 99% of the osteosarcoma patient samples exhibited less than 20% displacement of PT430 by methotrexate, and 88% had methotrexate displacement at less than half of the corresponding trimetrexate displacement of PT430 (*n* = 69). This is strong evidence that osteosarcoma harbors some level of intrinsic resistance to methotrexate due to impaired transport. Only nine of the 69 samples (13%) exhibited elevated peak PT430 levels suggestive of DHFR overexpression. These results suggest that methotrexate resistance is a result of impaired transport via the reduced folate carrier rather than DHFR overexpression. Given the evident intrinsic methotrexate resistance in osteosarcoma, evaluation of antifolate agents that do not rely on transport via the RFC is warranted for recurrent or refractory disease. Potentially, the degree of intrinsic methotrexate resistance could be determined at diagnosis and could help define individualized therapy for osteosarcoma.

Forty five of the 50 biopsy samples (90%) had an MTX : TMTX differential displacement ratio of <0.50; that is, displacement of PT430 by methotrexate was less than half of the displacement by trimetrexate. The human lymphoblast cell line CCRF-CEM is known to be sensitive to methotrexate and exhibited an MTX : TMTX differential displacement ratio of 0.99 (data not shown). Nine of 11 patient samples (82%) taken at time of definitive surgery exhibited methotrexate displacement at less than half of the corresponding trimetrexate displacement. Similarly, six of eight samples (75%) taken either at time of relapse or from metastatic disease had methotrexate displacement at less than half of trimetrexate displacement. In summary, functional methotrexate defects were observed in osteosarcoma regardless of sample type: biopsy, definitive resection, or relapse.

Since methotrexate enters the cell primarily through the reduced folate carrier, it could be plausibly concluded that the prevalence of impaired methotrexate transport in osteosarcoma is due to altered function of the reduced folate carrier. It has been previously reported that over 50% of osteosarcoma samples have decreased reduced folate carrier expression at time of diagnosis as determined by semiquantitative PCR [13]. The decreased reduced folate carrier expression was associated with inferior response to preoperative chemotherapy, but functional methotrexate transport was not assessed in these samples. 

Sequence alterations of the reduced folate carrier have been shown to occur in osteosarcoma. The functional significance of several reduced folate carrier sequence variants was evaluated by transfecting several different altered human reduce folate carrier fusion proteins into a reduced folate carrier-null hamster cell line [21] or null HeLa cell line [22]. Four of the altered hamster lines resulted in less effective transport of methotrexate as compared to wild type. Likewise, four of the altered HeLa lines resulted in either reduced or abolished methotrexate transport in comparison to wild type. It is expected that these sequence variants in human osteosarcoma cell lines would yield a similar result. Other reported alterations to the reduced folate carrier include multiple sequence variants in exons 2 and 3 [9], promoter methylation [23], and high-frequency splice variants [24]. 

Although therapeutically informative, functional transport assays are not likely to be prognostic given the high frequency of transport defects. Changes in reduced folate carrier expression level as opposed to sequence alterations may be prognostic as the relevance may be the mechanism of functional inactivation. It remains unclear why even at diagnosis, prior to methotrexate exposure, the methotrexate-reduced folate carrier transport pathway is functionally inactivated. Further characterization of the reduced folate carrier alterations may provide more insight into basic folate transport mechanisms and steps in osteosarcoma tumor pathogenesis.

## Figures and Tables

**Table 1:** Displacement of methotrexate and trimexate by PT430 grouped by sample type.

		All samples	Biopsy	Definitive surgery	Metastatic disease or relapse
% PT430 Displacement by methotrexate	*n*	69	50	11	8
	Range	0%–50.97%	0%–19.28%	0%–50.97%	1.13%–14.71%
	Median	3.10%	2.29%	5.81%	7.62%
	Average	4.88%	3.60%	8.89%	7.36%

% PT430 Displacement by trimetrexate	Range	3.27%–86.69%	3.27%–59.01%	6.84%–86.69%	12.48%–83.13%
	Median	21.05%	18.24%	30.50%	27.23%
	Average	24.66%	20.63%	32.81%	38.69%

MTX : TMTX Differential displacement ratio^a^	Range	0.00–3.60	0.00–3.60	0.00–0.95	0.04–0.64
	Median	0.15	0.12	0.22	0.13
	Average	0.25	0.24	0.27	0.25

^a^The MTX : TMTX differential displacement ratio represents the percentage of PT430 displaced by methotrexate in relation to the percentage of PT430 displaced by trimetrexate. For example, a sample with methotrexate displacement of 25% and trimetrexate displacement of 50% would have an MTX : TMTX differential displacement ratio of 0.50.
